# A nomogram for predicting post-stroke cognitive impairment no dementia in patients with first-ever mild ischemic stroke

**DOI:** 10.3389/fneur.2025.1618953

**Published:** 2025-08-19

**Authors:** Zhenjie Teng, Jing Feng, Xiaohua Xie, Tianyuan Guan, Jing Xu, Xin Jiang, Yanzhong Chang, Peiyuan Lv

**Affiliations:** ^1^Postdoctoral Innovation Practice Base of Hebei General Hospital, Shijiazhuang, China; ^2^Postdoctoral Research Station of Biology, Hebei Normal University, Shijiazhuang, China; ^3^Department of Neurology, Hebei General Hospital, Shijiazhuang, China; ^4^Hebei Provincial Key Laboratory of Cerebral Networks and Cognitive Disorders, Shijiazhuang, China; ^5^Department of Endocrinology, Hebei General Hospital, Shijiazhuang, China; ^6^Laboratory of Molecular Iron Metabolism, Key Laboratory of Molecular and Cellular Biology of Ministry of Education, College of Life Sciences, Hebei Normal University, Shijiazhuang, China; ^7^Hebei Key Laboratory of Animal Physiology, Biochemistry and Molecular Biology, Hebei Research Center of the Basic Discipline of Cell Biology, College of Life Sciences, Hebei Normal University, Shijiazhuang, China

**Keywords:** post-stroke cognitive impairment, mild ischemic stroke, cerebral small vessel disease, nomogram, prediction model

## Abstract

**Objective:**

To identify significant predictors and construct a validated nomogram for predicting post-stroke cognitive impairment no dementia (PSCIND) risk among first-ever mild ischemic stroke (MIS) patients.

**Methods:**

This retrospective cohort study analyzed 242 first-ever MIS patients categorized into normal cognitive (*n* = 137) and PSCIND (*n* = 105) groups. Comprehensive data encompassing demographic characteristics, laboratory parameters, cerebral small vessel disease (CSVD) imaging markers, neuropsychological assessments, and ischemic stroke lesion characteristics were collected. Predictor selection was conducted through least absolute shrinkage and selection operator (LASSO) regression analysis, followed by multivariable logistic regression for nomogram construction. Model performance was assessed through discrimination (area under the curve), calibration (calibration plots, Hosmer-Lemeshow test), and clinical utility (decision curve analysis).

**Results:**

Eight independent predictors were identified: age (OR = 1.060, 95% CI: 1.016–1.106), education level (OR = 0.917, 95% CI: 0.845–0.995), type 2 diabetes mellitus (OR = 9.407, 95% CI: 3.761–23.528), superoxide dismutase (OR = 0.951, 95% CI: 0.931–0.972), uric acid (OR = 1.006, 95% CI: 1.002–1.010), homocysteine (OR = 1.058, 95% CI: 1.027–1.091), strategic infarcts (OR = 4.566, 95% CI: 2.148–9.707), and severe CSVD burden (OR = 3.818, 95% CI: 1.842–7.911). The nomogram exhibited excellent discrimination (AUC = 0.886) accompanied by satisfactory calibration (Hosmer-Lemeshow *χ*^2^ = 14.542, *p* = 0.104). Decision curve analysis showed clinical utility across threshold probabilities of 6–100%.

**Conclusion:**

This validated nomogram incorporating clinical, biochemical, and neuroimaging biomarkers provides a robust tool for individualized PSCIND risk assessment in first-ever MIS patients, with potential to guide targeted interventions and cognitive monitoring.

## Introduction

Stroke remains a foremost global health challenge (1), with post-stroke cognitive impairment (PSCI) emerging as a predominant debilitating disabling neuropsychiatric sequelae (2, 3). A multicenter study demonstrated that up to 44% of stroke survivors develop PSCI within the first 6 months post-event (4, 5), imposing a substantial burden on long-term functional outcomes. PSCI is defined as cognitive deficits persisting for 3–6 months post-stroke, categorized into two clinical subtypes: post-stroke cognitive impairment no dementia (PSCIND) and post-stroke dementia (PSD) (5). As an early stage of PSCI, PSCIND is characterized by measurable cognitive decline without fulfilling dementia diagnostic criteria, thereby serving as a critical window for early intervention.

Despite preserved basic activities of daily living, individuals with PSCIND exhibit significantly compromised quality of life metrics (5). A meta-analysis synthesizing 23 prospective cohorts revealed a pooled PSCIND prevalence of 38% during the first post-stroke year (6). Alarmingly, approximately 25% of PSCIND cases progress to PSD in the absence of timely interventions, underscoring the urgency of early identification and management (7). Nevertheless, validated predictive tools for PSCIND remain conspicuously absent in clinical practice.

Ischemic stroke, constituting 62% of global stroke incidence (8), presents unique diagnostic and therapeutic challenges in PSCI management. Mild ischemic stroke (MIS), operationally defined by National Institutes of Health Stroke Scale (NIHSS) scores ≤5 (9, 10), represents more than half of incident ischemic cerebrovascular events (11). Notably, the subtle neurological deficits characteristic of MIS frequently result in systematic underassessment of cognitive domains, despite neuropsychological evidence indicating high PSCI prevalence in MIS patients (5, 12). This clinical oversight stems from the misconception that mild neurological symptoms equate to negligible cognitive consequences. While current evidence highlights MIS populations as priority targets for PSCI prevention, critical knowledge gaps persist regarding PSCIND predictors in first-ever MIS patients, with a paucity of validated prediction models tailored to this subgroup.

To address these challenges, this retrospective study systematically evaluates demographic, biochemical, and neuroimaging predictors of PSCIND in first-ever MIS patients. We aim to develop and validate a clinically actionable, visualization-enhanced prediction tool. This model seeks to enable individualized PSCIND risk assessment, thereby informing targeted interventions to mitigate cognitive decline and improve long-term outcomes in this vulnerable population.

## Methods

### Patients

This prognostic modeling study employed a retrospective cohort design using data from the Neurology Department of Hebei General Hospital (August 2019–December 2022). Inclusion criteria comprised: (a) age ≥50 years; (b) first-ever MIS (NIHSS score ≤5) confirmed by neuroimaging; (c) availability of complete clinical variables and neuropsychological assessments. Exclusion criteria were rigorously applied to minimize confounding: (a) transient ischemic attack or pre-stroke cognitive impairment (documented by medical records or caregiver reports); (b) comorbidities potentially influencing cognitive assessments, such as active epilepsy/seizure disorders, traumatic brain injury history and major psychiatric disorders (e.g., schizophrenia, severe depression); (c) unavailable 3–6 month follow-up data in the institutional database; (d) recurrent cerebrovascular events during 3–6 month follow-up period; (e) diagnosis of PSD during the 3–6 month follow-up period; (f) anti-dementia medications were used during the 3–6 month follow-up period. The flow chart of participant selection is illustrated in Supplementary Figure S1. The diagnostic prediction model was developed and reported in full compliance with the Transparent Reporting of a multivariable prediction model for Individual Prognosis Or Diagnosis (TRIPOD) statement, specifically following the Type 1b framework (development using existing data from a single-center retrospective cohort) (13). Conducted in compliance with the Declaration of Helsinki, this study obtained ethical clearance from the Ethics Committee of Hebei General Hospital (No.2025-LW-0124).

### Evaluation of post-stroke cognitive impairment no dementia

Standardized neuropsychological evaluations were administered to all participants, incorporating three validated measures: the Beijing-adapted Montreal Cognitive Assessment,^1^ alongside assessments of basic (BADL) and instrumental (IADL) activities of daily living. Initial cognitive and functional evaluations were conducted within 14 days post-MIS onset, with follow-up assessments scheduled at 3–6 months. Cognitive impairment was defined using education-adjusted MoCA cutoff criteria based on Chinese normative data. Cognitive impairment thresholds were defined according to Chinese normative data with education-stratified cutoffs: ≤13 for illiterate individuals, ≤19 for those with 1–6 years of educational attainment, and ≤24 for individuals with 7 or more years of education (14). PSCIND diagnosis required: (a) MoCA scores below education-adjusted thresholds at 3–6 month follow-up; (b) no or mild impairment in both BADL and IADL, thereby excluding dementia (2, 15, 16). Based on these criteria, first-ever MIS patients were stratified into two groups: PSCIND and normal cognitive group.

### Predictor selection and data acquisition

Candidate predictors were retrospectively collected from electronic medical records and structured clinical assessments. Demographic variables included age, sex, educational attainment, and body mass index. Clinical parameters comprised smoking/alcohol use history, type 2 diabetes mellitus (T2DM) diagnosis, hypertension status, coronary heart disease history, and admission systolic/diastolic blood pressure (SBP/DBP). Fasting venous blood samples (≥8 h) were analyzed for metabolic, inflammatory, oxidative stress, and coagulation biomarkers, including fasting plasma glucose (FPG), lipid profile components [triglycerides, total cholesterol, high-density, low-density and very low density lipoprotein cholesterol, apolipoprotein A, apolipoprotein B, and lipoprotein (a)], superoxide dismutase (SOD), homocysteine (Hcy), and uric acid (UA), and fibrinogen. Neurological evaluations incorporated NIHSS for acute stroke severity and the modified Rankin Scale (mRS) for functional outcomes. Ischemic stroke subtypes were classified into three categories based on neuroimaging characteristics: (1) multiple infarcts (≥2 distinct vascular territories), (2) strategic infarcts (functionally critical regions including the thalamus, caudate nucleus, frontal cortex, medial temporal lobe, and angular gyrus), or (3) small-artery occlusion (subcortical lesions <20 mm). Cerebral small vessel disease (CSVD) burden was quantified using a validated total CSVD score (range: 0–4) derived from four neuroimaging markers, as detailed in our prior studies (17). A threshold score >2 defined severe CSVD burden.

### Statistical analysis

Statistical analyses were performed using SPSS 26.0 (IBM Corp., Armonk, NY) and R 4.2.3 (R Foundation for Statistical Computing, Vienna, Austria). Normally distributed continuous variables were summarized as mean ± standard deviation, non-normally distributed variables as median (interquartile range), and categorical variables as frequencies (%). Group comparisons employed independent t-tests for parametric data, Mann–Whitney U tests for nonparametric data, and χ^2^ tests for categorical variables, based on distributional assumptions. To mitigate multicollinearity and overfitting, least absolute shrinkage and selection operator (LASSO) regression (“glmnet” package) identified optimal predictors for PSCIND in first-ever MIS patients. Variables retained through LASSO regression were entered into multivariable logistic regression to develop the final predictive model, which was graphically represented through a static nomogram (“rms” package) and an interactive web-based visualization tool (“DynNom” and “shiny” packages) deployed as a Shiny application. Model evaluation encompassed four key assessments: (1) discrimination capacity measured by receiver operating characteristic (ROC) curve analysis [quantified by area under the curve (AUC)]; (2) calibration accuracy evaluated through calibration plots with Hosmer-Lemeshow testing; (3) clinical applicability determined via decision curve analysis (DCA); and (4) predictive overall performance quantified by the Brier score (<0.25 indicating superior performance). Internal validation with 2000 bootstrap resamples enhanced model reliability, with statistical significance set at two-tailed *p* < 0.05.

## Results

### Participants characteristics

The study cohort comprised 242 first-ever MIS patients with a median age of 65 years (male predominance: 67.8%). Participants were stratified into normal cognitive (*n* = 137) and PSCIND (*n* = 105) groups. Comparative analysis demonstrated that PSCIND patients were significantly older, had lower educational attainment, and exhibited higher prevalence of hypertension and T2DM history. Biochemical analyses identified distinct biomarker patterns, with the PSCIND group displaying reduced SOD levels concomitant with elevated UA, fibrinogen, and Hcy levels relative to cognitively normal subjects. Neuroimaging assessments revealed significant between-group differences in lesion distribution: PSCIND patients demonstrated greater prevalence of multiple infarcts (34.3% vs. 21.6%), strategic infarcts (63.8% vs. 39.4%), and severe CSVD burden (57.1% vs. 24.8%), alongside reduced incidence of small artery occlusion subtype (44.8% vs. 61.3%) compared to cognitively normal counterparts. All intergroup comparisons reached statistical significance (*p* < 0.05), with comprehensive comparative data detailed in Table 1.

**Table 1:** Characteristics of the study patients with first-ever MIS between normal cognitive group and PSCIND group.

Variable	Total (*n* = 242)	Normal cognitive group (*n* = 137)	PSCIND group (*n* = 105)	*p* value
Age, median (IQR), year	65 (58–73)	63 (56–71)	68 (63–75)	< 0.001*
Sex (male), *n* (%)	164 (67.8)	88 (64.2)	76 (72.4)	0.179
Education, median (IQR), year	9 (6–14)	12 (9–15)	9 (6–12)	0.001*
Body mass index, median (IQR), kg/m^2^	25.0 (22.9–27.6)	25.0 (23.0–27.4)	25.0 (22.9–27.7)	0.895
Smoking, *n* (%)	108 (44.6)	61 (44.5)	47 (44.8)	0.971
Alcohol use, *n* (%)	71 (29.3)	38 (27.7)	33 (31.4)	0.532
Hypertension, *n* (%)	148 (61.2)	75 (54.7)	73 (69.5)	0.019*
SBP, median (IQR), mmHg	148 (136–162)	147 (136–162)	148 (136–163)	0.976
DBP, mean ± SD, mmHg	84.6 ± 11.9	85.2 ± 11.8	83.8 ± 12.1	0.366
T2DM, *n* (%)	70 (28.9)	32 (23.4)	38 (36.2)	0.029*
Coronary heart disease, *n* (%)	29 (12.0)	16 (11.7)	13 (12.4)	0.868
FPG, median (IQR), mmol/L	5.25 (4.72–6.75)	5.29 (4.74–6.76)	5.16 (4.72–6.71)	0.678
TG, median (IQR), mmol/L	1.29 (0.96–1.78)	1.28 (0.95–1.73)	1.31 (0.96–1.89)	0.795
TC, median (IQR), mmol/L	4.57 (3.79–5.23)	4.59 (3.92–5.22)	4.38 (3.64–5.26)	0.234
HDL-C, median (IQR), mmol/L	1.06 (0.91–1.24)	1.07 (0.91–1.26)	1.03 (0.91–1.20)	0.229
LDL-C, median (IQR), mmol/L	2.99 (2.40–3.50)	3.05 (2.50–3.51)	2.85 (2.35–3.48)	0.145
VLDL-C, median (IQR), mmol/L	0.47 (0.28–0.65)	0.46 (0.28–0.63)	0.48 (0.28–0.70)	0.608
ApoA1, median (IQR), g/L	1.18 (1.03–1.36)	1.20 (1.04–1.37)	1.17 (1.01–1.36)	0.297
ApoB, median (IQR), g/L	0.81 (0.69–0.96)	0.83 (0.70–0.97)	0.79 (0.68–0.94)	0.252
Lp (a), median (IQR), mg/L	178 (100–313)	171 (95–317)	186 (103–310)	0.648
SOD, median (IQR), U/mL	142 (129–156)	150 (137–162)	133 (120–143)	<0.001*
UA, median (IQR), μmol/L	296 (244–352)	281 (225–338)	308 (267–372)	0.001*
Fibrinogen, median (IQR), g/L	2.85 (2.40–3.34)	2.72 (2.26–3.16)	3.02 (2.66–3.52)	<0.001*
Hcy, median (IQR), umol/L	14.3 (11.8–19.5)	12.9 (10.6–17.7)	16.8 (13.3–22.8)	<0.001*
NIHSS, median (IQR), score	2 (1–3)	2 (1–3)	2 (1–3)	0.514
MRS, median (IQR), score	2 (1–3)	2 (1–3)	2 (2–3)	0.099
Multiple infarcts, *n* (%)	66 (27.3)	30 (21.6)	36 (34.3)	0.032*
Strategic infarcts, *n* (%)	121 (50.0)	54 (39.4)	67 (63.8)	<0.001*
Small-artery occlusion, *n* (%)	131 (54.1)	84 (61.3)	47 (44.8)	0.010*
Severe CSVD burden, *n* (%)	94 (38.8)	34 (24.8)	60 (57.1)	<0.001*

PSCIND, post-stroke cognitive impairment no dementia; IQR, interquartile range; SD, standard deviation; SBP, systolic blood pressure; DBP, diastolic blood pressure; T2DM, type 2 diabetes mellitus; FPG, fasting plasma glucose; TG, triglyceride; TC, total cholesterol; HDL-C, high density lipoprotein cholesterol; LDL-C, low density lipoprotein cholesterol; VLDL-C, very low density lipoprotein cholesterol; ApoA1, apolipoprotein A1; ApoB, apolipoprotein B; Lp (a), lipoprotein (a); SOD, superoxide dismutase; UA, uric acid; Hcy, Homocysteine; NIHSS, National Institutes of Health Stroke Scale; mRS, modified Rankin Scale. *Denotes significance at a *p* value of <0.05.

### Predictor selection for predictive model

**Figure 1:**
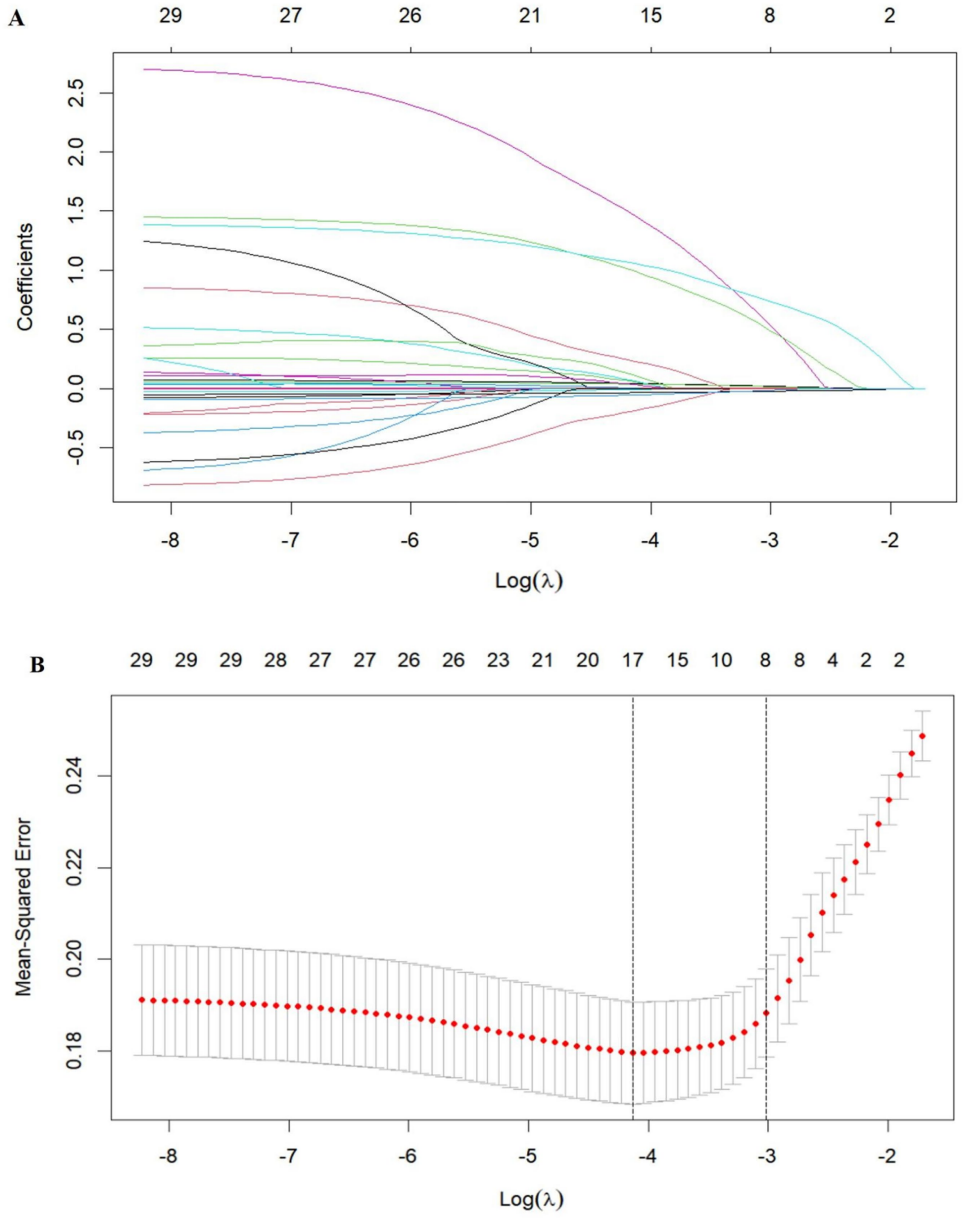
Variable selection by LASSO regression model. **(A)** LASSO coefficient profiles of the 30 variables. **(B)** Eight variables with nonzero coefficients were selected by optimal *λ*. The two dotted vertical lines were drawn at the optimal scores by minimum criteria and 1 standard error criteria.

All 30 candidate variables listed in Table 1 were subjected to LASSO regression analysis for PSCIND predictor identification. Figure 1A illustrates the coefficient trajectories across varying regularization parameters, demonstrating progressive feature shrinkage as *λ* increases. The optimal regularization parameter was determined through 10-fold cross-validation (Figure 1B), where model selection occurred at the λ value corresponding to the minimum deviance plus one standard error (right vertical dashed line). This analytical approach ultimately selected eight predictors retaining non-zero coefficients: age, education level, T2DM status, SOD levels, UA levels, Hcy levels, strategic infarcts, and severe CSVD burden.

### Predictive model development and visualization

A multivariablelogistic regression model incorporating eight LASSO-selected predictors (age, education level, T2DM, SOD, UA, Hcy, strategic infarcts, and severe CSVD burden) was established to assess PSCIND risk in first-ever MIS patients. The analysis demonstrated significant associations between these predictors and PSCIND in Table 2: age (OR = 1.060, 95% CI: 1.016–1.106; *p* = 0.007), education level (OR = 0.917, 95% CI: 0.845–0.995; *p* = 0.037), T2DM (OR = 9.407, 95% CI: 3.761–23.528; *p* < 0.001), SOD (OR = 0.951, 95% CI: 0.931–0.972; *p* < 0.001), UA (OR = 1.006, 95% CI: 1.002–1.010; *p* = 0.005), Hcy (OR = 1.058, 95% CI: 1.027–1.091; *p* = 0.013), strategic infarcts (OR = 4.566, 95% CI: 2.148–9.707; *p* < 0.001), and severe CSVD burden (OR = 3.818, 95% CI: 1.842–7.911; *p* < 0.001).

**Table 2:** Multivariate logistic regression analysis of the associated predictors for the risk of PSCIND in patients with first-ever MIS.

Variable	*β*	SE	Wald *χ*^2^	*p* value	OR	95% CI
Age	0.059	0.022	7.350	0.007	1.060	1.016–1.106
Education	−0.087	0.041	4.355	0.037	0.917	0.845–0.995
T2DM	2.241	0.468	22.967	<0.001	9.407	3.761–23.528
SOD	−0.050	0.011	20.651	<0.001	0.951	0.931–0.972
UA	0.006	0.002	7.861	0.005	1.006	1.002–1.010
Hcy	0.057	0.015	13.602	0.013	1.058	1.027–1.091
Strategic infarcts	1.519	0.385	15.578	<0.001	4.566	2.148–9.707
Severe CSVD burden	1.340	0.372	12.988	<0.001	3.818	1.842–7.911

**Figure 2:**
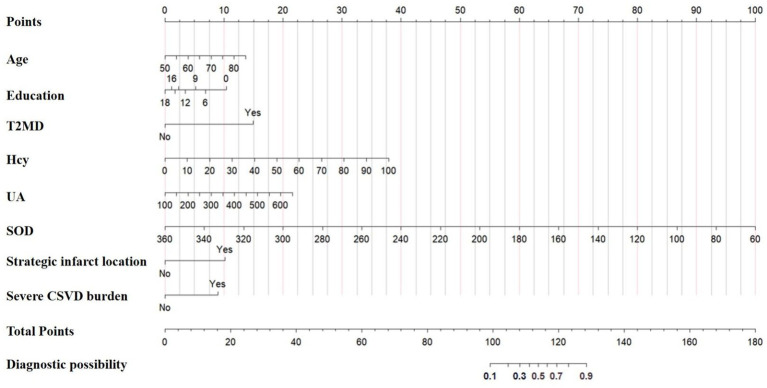
Nomogram for the prediction of the probability of PSCIND risk in patients with first-ever MIS.

**Figure 3:**
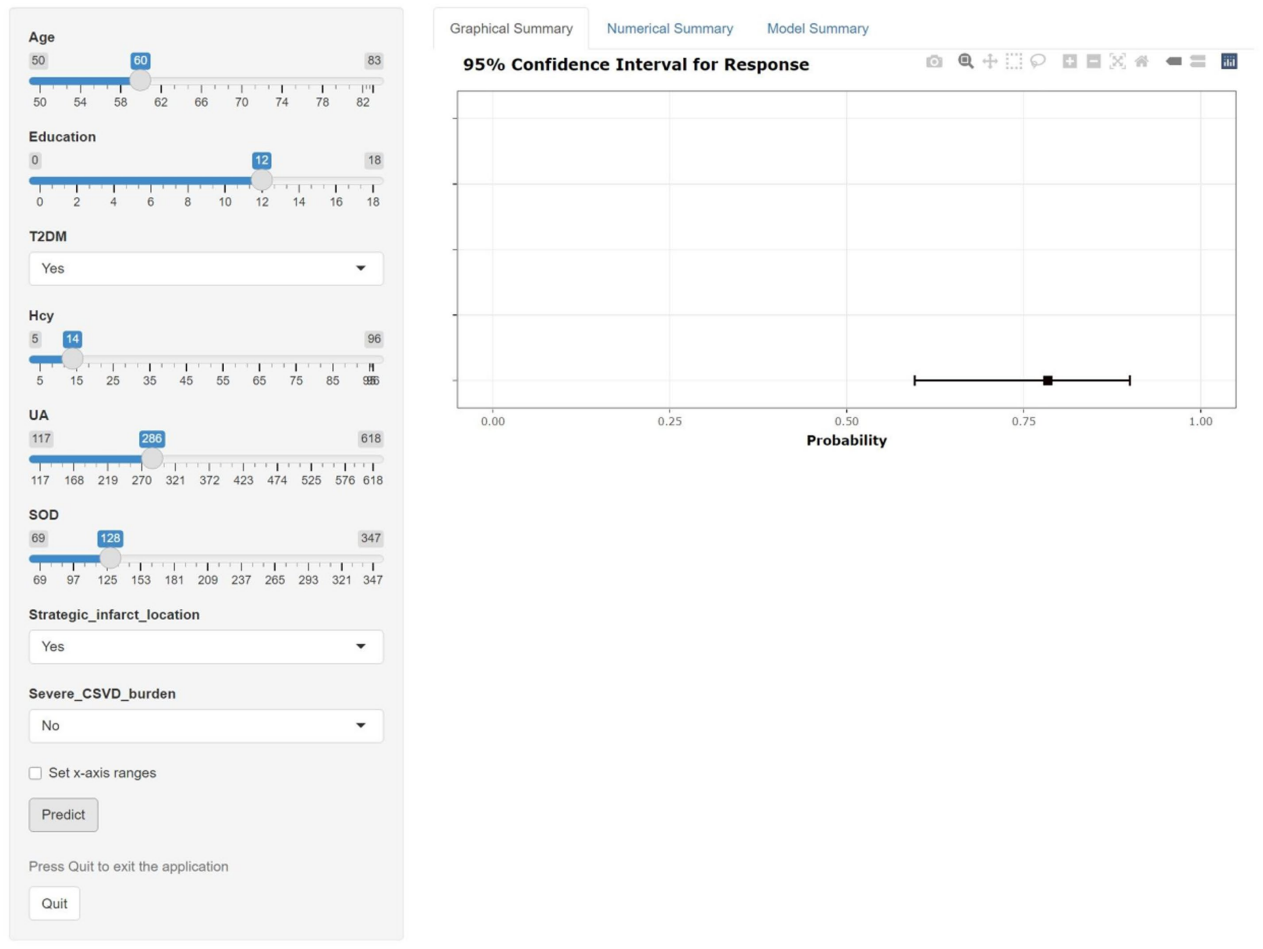
Dynamic nomogram for the prediction of the probability of PSCIND risk in patients with first-ever MIS. An example for predicting the probability of having PSCIND risk in a first-ever MIS with online dynamic nomogram. A 60-year-old patient with 12-year education, T2DM history, hcy 14 μmol/L, SOD 128 U/mL, UA 286 μmol/L, thalamic infarct, and CSVD score of 2 demonstrates a predicted 78.4% probability of developing PSCIND.

A clinical nomogram incorporating these predictors was constructed to estimate individualized PSCIND risk (Figure 2). Each predictor is assigned points on the upper axis, with total scores corresponding to predicted probabilities on the lower scale through vertical alignment. To enhance clinical utility, we developed an interactive web-based nomogram.^2^ For instance, a 60-year-old patient with 12-year education, T2DM history, Hcy 14 μmol/L, SOD 128 U/mL, UA 286 μmol/L, thalamic infarct, and CSVD score of 2 would have a 78.4% predicted probability of developing PSCIND (Figure 3).

### Predictive model validation

**Figure 4:**
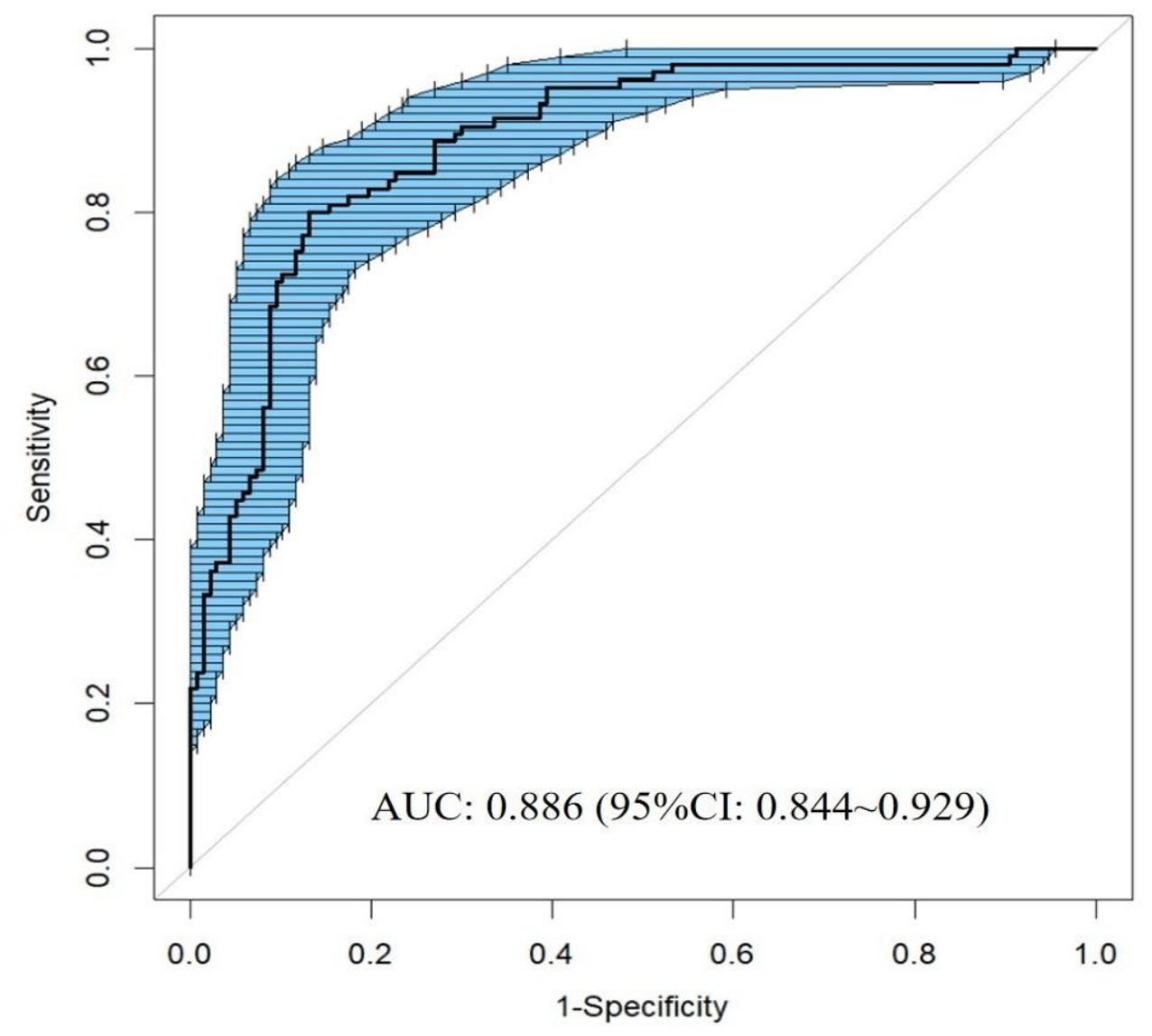
ROC curve of the nomogram for predicting PSCIND in patients with first-ever MIS.

**Figure 5:**
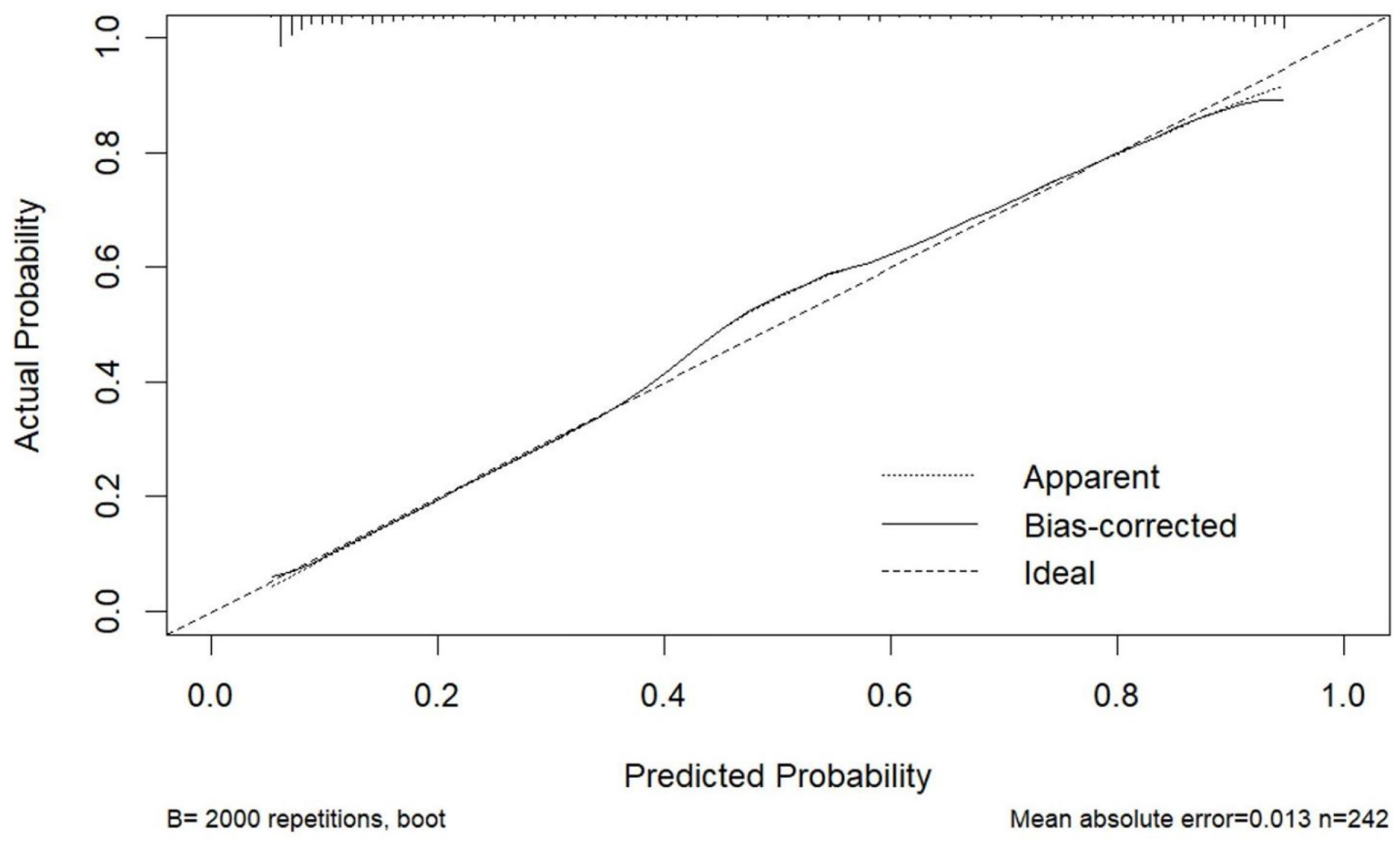
Calibration curve of the nomogram for predicting PSCIND in patients with first-ever MIS.

**Figure 6:**
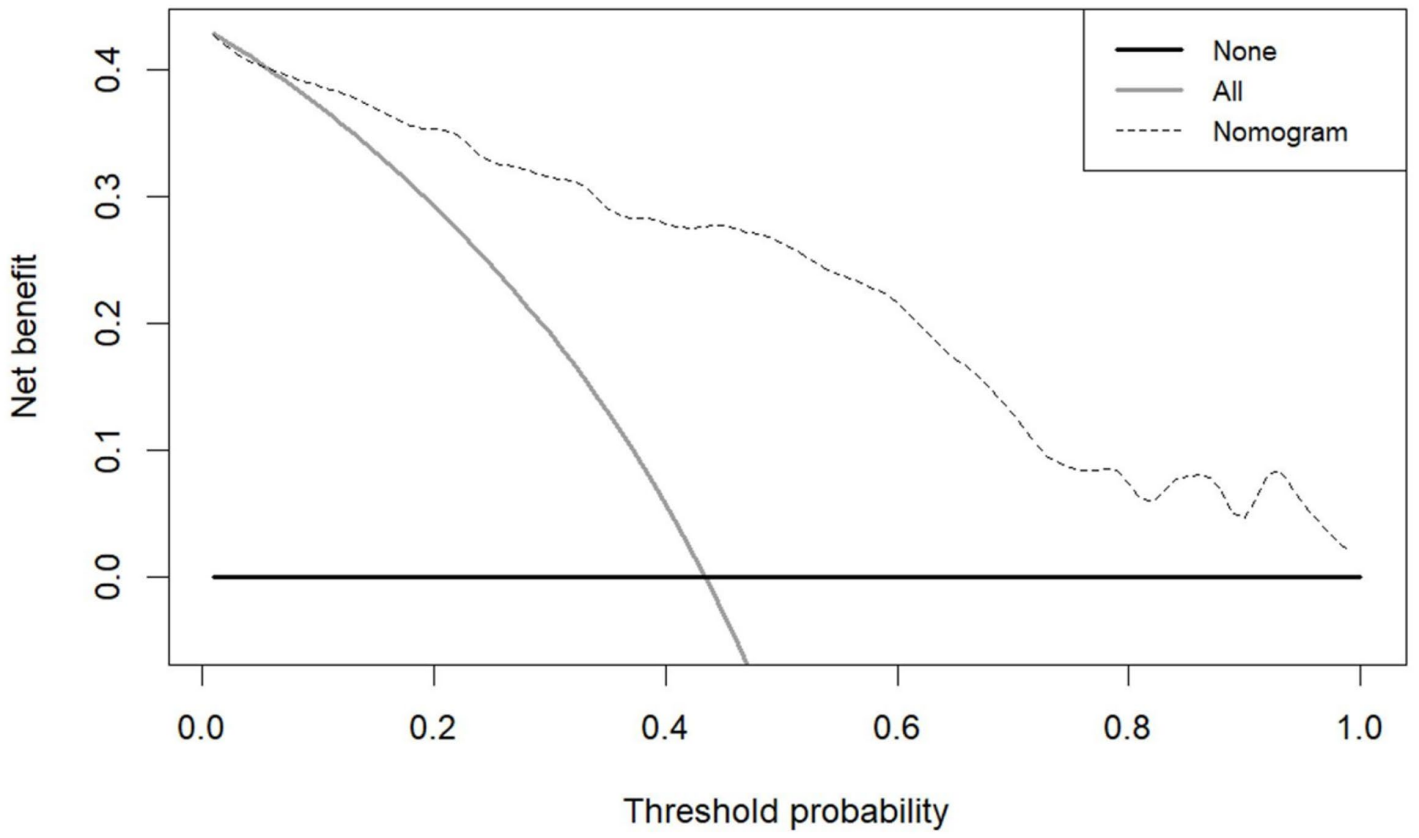
DCA of the nomogram for predicting PSCIND in patients with first-ever MIS.

The model demonstrated excellent discriminative capacity through ROC curve analysis with 2000 bootstrap resampling validations, achieving an AUC of 0.886 (95% CI: 0.844–0.929) (Figure 4). Calibration accuracy was confirmed via bootstrap-corrected calibration curves (2000 iterations) and Hosmer-Lemeshow test (*χ*^2^ = 14.542, *p* = 0.104), demonstrating strong concordance between predicted and observed probabilities (Figure 5). The model achieved a Brier score of 0.135 (<0.25 threshold), confirming superior overall predictive accuracy. DCA demonstrated enhanced clinical utility across 6–100% threshold probabilities, outperforming alternative strategies in net clinical benefit (Figure 6).

## Discussion

This study revealed a 43.4% prevalence of PSCIND in first-ever MIS patients and developed a validated clinical prediction model incorporating eight predictors: age, education level, T2DM, SOD, UA, Hcy, strategic infarcts, and severe CSVD burden. The model demonstrated robust discriminative capacity, excellent calibration, and clinical applicability, providing clinicians with a practical tool for early risk stratification in this population.

Advanced age and lower education emerged as critical demographic predictors, with each additional year of age (50–85 years) increasing PSCIND risk by 6% and each year of education reducing risk by 8%. These findings align with global evidence on socioeconomic and biological determinants of post-stroke cognitive decline (2, 18). Notably, T2DM exhibited the strongest predictive value (OR = 9.407), aligning with meta-analyses identifying diabetes as a principal risk modulator for PSCI (4).

Blood-based biomarker research has significantly improved PSCI diagnostic accuracy (19–21). Hcy has garnered significant attention as a readily measurable biomarker linked to neurological disorders, with emerging evidence supporting its role in PSCI and other cognitive impairments (22). A meta-analysis highlighted elevated Hcy as a potential early diagnostic marker for PSCI (23), aligning with our findings that higher Hcy levels significantly predicted PSCIND risk in first-ever MIS. However, a multicenter prospective study in MIS (NIHSS≤3) and transient ischemic attack patients reported no association between Hcy and 3-month PSCI incidence, though a gender-specific risk emerged at 12 months (24). These discrepancies may stem from cohort heterogeneity (e.g., stroke severity thresholds) and inadequate stratification of PSCI subtypes (e.g., PSCIND vs. dementia), underscoring the need for extended follow-up and subgroup analyses by sex/age in future studies.

The prognostic utility of UA exhibits ongoing controversy: while multiple studies have identified elevated serum UA levels as a sensitive predictor for PSCI development (25, 26), others paradoxically suggest a protective effect of hyperuricemia against cognitive decline (27). Data from the China National Stroke Registry-III revealed a U-shaped relationship between UA levels and PSCI risk in males (28), yet a meta-analysis found no diagnostic utility for UA in PSCI prediction (29). Notably, prior studies often neglected PSCI subtype stratification, ischemic lesion assessment, and critical imaging markers like CSVD burden. By systematically accounting for these factors, our study identified elevated UA as a significant PSCIND predictor in first-ever MIS patients. This finding underscores the necessity of contextualizing UA measurements within broader cerebrovascular evaluations, though large-scale multicenter validation is warranted.

SOD, a pivotal antioxidant enzyme, indirectly reflects the systemic capacity to eliminate oxygen free radicals and is crucial in mitigating oxidative stress (30, 31). Experimental and clinical data demonstrate that reduced SOD activity contributes to cognitive disorder pathogenesis through chronic oxidative damage, vascular endothelial impairment, and blood–brain barrier compromise (32–34). Despite its pathophysiological significance, SOD has been insufficiently investigated in PSCI research. Although a previous study identified an association between low SOD levels and PSCI risk in MIS patients (NIHSS≤8), the investigation omitted critical exclusion criteria (e.g., recurrent strokes potentially confounding cognitive trajectories) and neglected PSCI subtype stratification (34). Our study newly establishes diminished SOD activity as an independent predictor (OR = 0.951), mechanistically implicating oxidative stress in PSCIND pathogenesis while highlighting antioxidant supplementation as a plausible therapeutic strategy-an innovative dimension previously absent in predictive models.

The neuroanatomical distribution of ischemic stroke lesions significantly influences the risk of PSCI (35). Strategic infarcts, defined as lesions affecting brain regions critical for higher cortical functions-including the thalamus, caudate nucleus, frontal cortex, medial temporal lobe (including the hippocampus), and angular gyrus-have been identified as independent PSCI risk factors (36, 37). Previous studies have demonstrated the particular vulnerability of left middle frontal gyrus, anterior thalamic nuclei, and left angular gyrus in PSCI pathogenesis (37, 38). A study synthesizing data from 12 acute ischemic stroke cohorts further highlighted the strong association between PSCI and infarcts in the left frontotemporal lobe, left thalamus, and right parietal lobe (36). Our findings corroborate this evidence, identifying strategic infarcts as significant predictors of PSCIND in MIS patients (OR = 4.566). Nevertheless, the lack of granular anatomical stratification of infarct locations in our analysis highlights the need for future investigations to delineate region-specific contributions to cognitive outcomes.

Compared to isolated CSVD imaging markers, CSVD burden assessment provides a more comprehensive evaluation of cerebrovascular injury and better aligns with cognitive diagnostic frameworks (39, 40). Sung et al. (41) demonstrated that severe baseline CSVD burden (total score ≥3) independently predicts 1-year PSCI incidence in first-ever MIS patients-a finding paralleling our observed association between severe CSVD burden and elevated PSCIND risk (OR = 3.818). However, Du et al. (42) proposed an indirect relationship, where CSVD burden influences PSCI via disrupted brain network connectivity rather than direct effects. A multicenter prospective study substantiated the prognostic value of CSVD burden, correlating baseline scores with impaired executive function, attention, and visuospatial abilities at 12-month follow-up (43). These collective findings underscore the necessity of incorporating CSVD burden-rather than isolated markers-into PSCIND prediction models for first-ever MIS patients.

This study introduces three principal advancements: (1) The first dedicated model for first-ever MIS patients; (2) Incorporation of novel oxidative stress (SOD) and neuroimaging (CSVD burden) biomarkers; (3) Development of an interactive web-based nomogram enhancing clinical translation. However, several limitations warrant consideration. First, its retrospective design and single-center data may introduce selection bias and limit generalizability. Second, the moderate sample size restricts statistical power for subgroup analyses. Third, potential attrition bias must be acknowledged, as 76 patients (23.9% of eligible cohort) were lost to follow-up. Fourth, although CSVD burden was incorporated as a composite metric, constituent markers were not individually assessed. Fifth, Broad “strategic infarct” categorization obscures region-specific effects, necessitating finer anatomical stratification in future work. Prospective multi-center studies with larger cohorts and extended follow-up periods are needed to validate these findings and explore temporal biomarker dynamics.

## Conclusion

We developed and validated an eight-predictor nomogram integrating demographic, biochemical, and neuroimaging parameters to assess PSCIND risk in first-ever MIS patients. The model’s discriminative accuracy and dynamic visualization interface facilitate early identification of high-risk individuals, enabling targeted interventions to mitigate cognitive decline. While demonstrating immediate clinical value, future large-scale multicenter studies integrating multi-omics approaches and neuroimaging biomarkers are warranted to enhance clinical implementation potential and elucidate underlying pathophysiological mechanisms.

## Data Availability

The original contributions presented in the study are included in the article/Supplementary material, further inquiries can be directed to the corresponding author/s.

## Footnotes

^1^MoCA; www.mocatest.org.

^2^Accessible at https://tengzhenjie.shinyapps.io/DynNomapp4/.
