# Supplementary material for: A nomogram for predicting post-stroke cognitive impairment no dementia in patients with first-ever mild ischemic stroke
Source: Front Neurol. 2025 Aug 19;16:1618953. doi: 10.3389/fneur.2025.1618953 (PMC12402937; doi:10.3389/fneur.2025.1618953)
Supplement: Supplementary file 2 [file Table_2.DOC]

**Supplementary Table S1 Anatomic Distribution of Strategic Infarcts in patients with first-ever MIS**

| Anatomic Location | **Total**  **（n=121）** | **Normal cognitive group**  **（n=54）** | **PSCIND group （n=67）** |
| --- | --- | --- | --- |
| thalamus  caudate nucleus  frontal cortex  medial temporal lobe  angular gyrus | 68 （56.2）  3 （2.5）  19 （15.7）  15 （12.4）  16 （13.2） | 31（57.4）  0 （0.0）  8 （14.8）  6 （11.1）  9 （16.7） | 37（55.2）  3 （4.5）  11（16.4）  9 （13.4）  7 （10.5） |
