# Supplementary figures and images for: A nomogram for predicting post-stroke cognitive impairment no dementia in patients with first-ever mild ischemic stroke
Source: Front Neurol. 2025 Aug 19;16:1618953. doi: 10.3389/fneur.2025.1618953 (PMC12402937; doi:10.3389/fneur.2025.1618953)

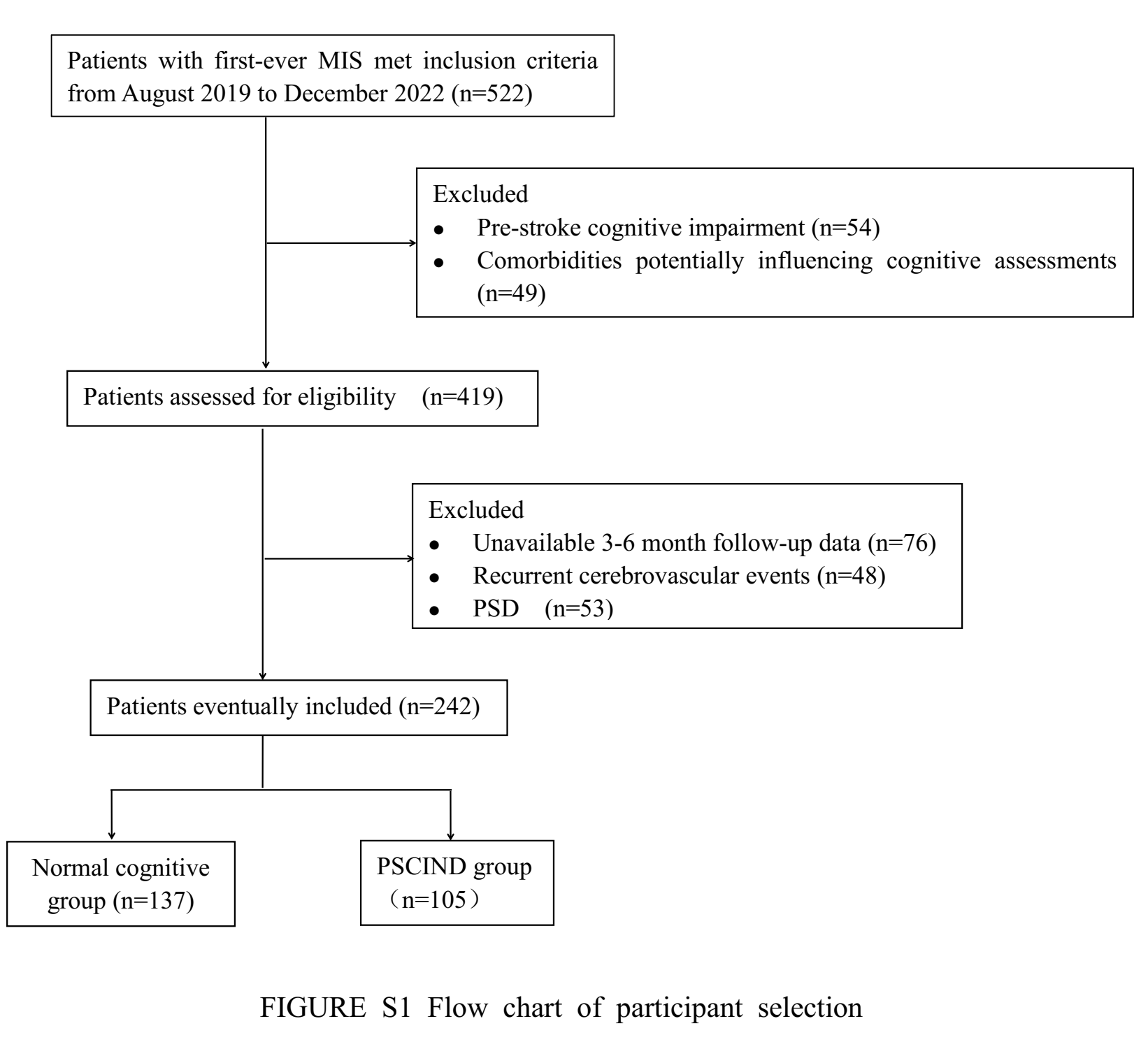

Supplement: Supplementary file 3 [file Image_1.TIFF]
